# Woman's True Rights

**Published:** 1873-04

**Authors:** 


*Woman's True Rights.*—In a prominent daily paper we find the following under this heading:

"The Pennsylvania Dental College of Philadelphia is in a state of great internal commotion, in consequence of the exclusion from the second course of three ladies who had been matriculated and attended the first course. The dissatisfaction of the male students threatened greatly to diminish their attendance, and the authorities thus yielded to the pecuniary exigencies of the case."

Two of the ladies referred to in this communication have already matriculated in the Baltimore College of Dental Surgery for the session of 1873 and 1874--the faculty of this institution, from past experience, having no objection whatever to receiving female students.

Why the male students of the Pennsylvania College should object to those of the opposite sex, we cannot understand—as it has been entirely different in the Baltimore College during the past two sessions; where respect and consideration, instead of enmity, have characterized the behaviour of the male portion of the class towards the female members.

We are not so uncharitable as to ascribe this opposition on the part of Northern students to a want of that courtesy and respect so justly woman's due, and which have been manifested by Southern dental students in the Baltimore College, (where a great majority of the class are from the South,) thereby maintaining their claim to be gentlemen, but rather to the fear on the part of Northern men that perhaps their domain of practice is about to be invaded by the opposite sex.

